# A Re-Audit Assessing the Standardisation of Admission Blood Tests for Patients on Norbury House (PICU)

**DOI:** 10.1192/bjo.2022.501

**Published:** 2022-06-20

**Authors:** Raminderjit Kaur, Sarah Sargent, Hari Shanmugaratnam, Mark Winchester

**Affiliations:** Midlands Partnership NHS Foundation Trust, Stafford, United Kingdom

## Abstract

**Aims:**

Assess if variation exists for routine blood tests performed on admission, evaluate compliance with MPFT guidelines when performing routine admission blood tests, compare results with the previous audit completed in October/November 2020 and identify strategies to improve and standardise admission blood tests.

**Methods:**

Retrospective blood result data were collected for all admissions to Norbury House (PICU) at St George's Hospital in Stafford over a two month period. For the original audit, this was between October and November 2020, following which a staff education programme raised awareness of trust guidelines regarding admission blood tests. This was then re-audited in May and June 2021 to assess its impact. Patients transferred from acute wards were included but repeat admissions were omitted. Data analysis was completed through Microsoft Excel.

**Results:**

17 patients were included in the audit in October and November 2020 while 13 patients were included for the May and June 2021 audit. As per trust guidelines, the number of patients having the appropriate admission blood tests increased to 69% in 2021. Certain mandatory blood tests were requested far more regularly such as TFTs increasing from 71% in 2020 to 100% in 2021. Other vital blood tests on admission also increased substantially, such as Glucose increasing from 6% of admissions in 2020 to 69% in 2021 and Prolactin increasing from 77% in 2020 to 100% in 2021. All mandatory blood tests either increased in frequency or maintained a 100% completion rate, with the exception of Calcium which decreased slightly from 94% in 2020 to 92% in 2021.

In the 2020 audit, unnecessary blood tests were requested for 88% of patients which was reduced substantially to just 21% of admissions in the 2021 audit. The total number of unnecessary tests also greatly reduced from 23 tests in total in 2020 to 3 in 2021.

**Conclusion:**

It is vital that patients being admitted to the PICU have the appropriate blood tests completed on admission, as they may be prescribed psychotropic medication that impacts their physical health and may have been self-neglecting prior to admission. Although the audit shows that the interventions completed following the last review have been hugely successful in improving compliance with trust guidelines and reducing waste of NHS resources, there is still significant room for improvement through the continual education of staff. This should then be re-audited again in Spring 2022 to ensure that the improvement continues.

